# Epigenetics and *Helicobacter pylori*

**DOI:** 10.3390/ijms23031759

**Published:** 2022-02-03

**Authors:** Rosanna Capparelli, Domenico Iannelli

**Affiliations:** Department of Agricultural Sciences, University of Naples “Federico II”, Via Università, 100-Portici, 80055 Naples, Italy

**Keywords:** *Helicobacter pylori*, epigenetics, cancer, *Helicobacter pylori* and *Epstein-Barr* virus co-infection

## Abstract

Epigenetics regulates gene expression, cell type development during differentiation, and the cell response to environmental stimuli. To survive, bacteria need to evade the host immune response. Bacteria, including *Helicobacter pylori* (*Hp*), reach this target epigenetically, altering the chromatin of the host cells, in addition to several more approaches, such as DNA mutation and recombination. This review shows that *Hp* prevalently silences the genes of the human gastric mucosa by DNA methylation. Epigenetics includes different mechanisms. However, DNA methylation persists after DNA replication and therefore is frequently associated with the inheritance of repressed genes. Chromatin modification can be transmitted to daughter cells leading to heritable changes in gene expression. Aberrant epigenetic alteration of the gastric mucosa DNA remains the principal cause of gastric cancer. Numerous methylated genes have been found in cancer as well as in precancerous lesions of *Hp*-infected patients. These methylated genes inactivate tumor-suppressor genes. It is time for us to complain about our genetic and epigenetic makeups for our diseases.

## 1. Introduction

Epigenetics regulates gene expression, cell type differentiation during development, and the cell response to environmental stimuli. Consequently, genetics and epigenetics complement each other. To infect the host and survive, bacteria need to evade the immune response of the host. Bacteria reach this target epigenetically, altering the chromatin and the transcriptional program of the host cells through DNA methylation, histone modification, noncoding RNA, and RNA splicing factors, all named as epimutations (heritable changes, which alter gene expression but do not affect DNA sequence) [1].

**Figure 1:**
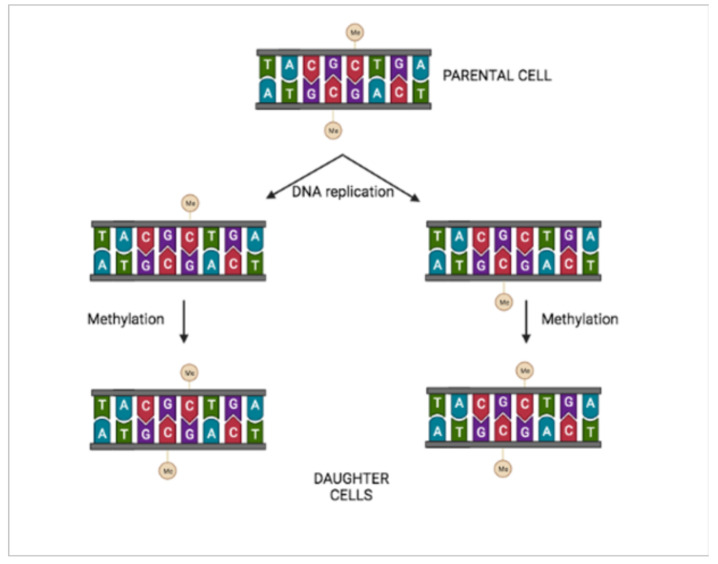
Inheritance of epigenetic information. Methyl groups of CG paired with methylated CG are conserved after DNA replication.

This review describes how hosts and pathogens coevolve, the unique properties of *Helicobacter pylori* (*Hp*), and the multiple interactions occurring between *Hp* and epigenetics. The review focuses on CpG methylation, which persists after DNA replication and is often associated with the inheritance of repressed genes [2] (Figure 1).

In this review, epigenetics is described as a means to control gene transcription. Yet, epigenetics is also involved in DNA repair, DNA replication, and cell division [1]. Understanding how epigenetics silences genes has already paved the way to therapeutic applications, such as reverting the function of specific methylated genes and preventing cancer development [3]. Improper use of antibiotics has brought the emergence of antibiotic resistance at a frightening level. So far, studies on the genetic origin of antimicrobial resistance in bacteria have focused exclusively on genetics. However, at present, there is clear evidence that epigenetic modification of the bacterial genome is a realistic new target [4].

## 2. Bacterial Infections

Pathogens coevolve in antagonism with their hosts: when a pathogen improves its ability to infect the host, reciprocally, the host sets up selection to evade the pathogen’s defense mechanism [5]. This process – known as the Red Queen dynamics [5]—generates and preserves biological diversity within populations [6,7]. Pathogens induce or silence host genes to aid their survival. Identifying signatures of coevolution (activated or repressed host genes following infection) helps recognize the host genes targeted by the pathogen and provides insights into the host-pathogen interactions. Reduced transcription of the host immunity genes often suggests that the pathogen inhibits the immune response. Conversely, increased gene transcription of the same genes suggests that the pathogen induces an excessive immune response. Generally, heat-killed bacteria stimulate fewer genes than live bacteria. When infected with heat-killed *Mycobacterium tuberculosis* (*M. tuberculosis*), macrophages show about 35% fewer genes with changed expression compared to those infected with live *M. tuberculosis* [8].

Following microbial infection, host cells mobilize the genes capable of evoking the most appropriate response against the pathogen. Antagonistically, the pathogen mobilizes the genes that can more efficiently contrast the host. The Red Queen dynamics force the host and pathogen to constantly adapt, evolve, and replicate in order to survive; briefly, to continuously evolve [9]. The Red Queen dynamics explains how the successful pathogens are those that more efficiently contrast host defense genes; at the same time, it reminds us that the success of the pathogen or of the host is ephemeral [10,11]. Yet, this input (that the success of the pathogen or the host is short-lived) is disregarded from genetic studies dealing with host protection against pathogens. In addition, the Red Queen dynamics theory demonstrates that hosts and pathogens coevolve antagonistically. However, the above studies rarely pay attention to the pathogen genotype; accordingly, the genetic diversity between strains of the same pathogen accounts—at least in part—for the low repeatability of these studies.

## 3. *Hp*: A Cosmopolitan Bacterium

*Hp* infects about half of the world’s population, and its infection can be long lasting. However, approximately 85% of the infected patients contract only a mild form of gastritis, 15% peptic ulcer, and less than 1% gastric cancer. Gastric cancer is the fifth most common and the third most deadly cancer worldwide [12]. The diverse diseases that *Hp* may cause depend on the virulence factors expressed by the bacterium (CagA, VacA, and BabA) host genetics (immune response), which may favor or obstacle *Hp* in its efforts to escape the host immune response, establish colonization, and induce disease. *Hp* is characterized by a high rate of genetic diversity derived from frequent recombination with different strains present in the same patient.

Genetic diversity alone represents a very important tool for the bacterium to evade recognition from host immunity. However, *Hp* can count on a multi-layered strategy to evade host immunity and establish chronic inflammation and long-term colonization of the host. *Hp* produces urease, which provides NH_3_ and CO_2_, indispensable to neutralize the high acidity of the stomach and permit the survival of the bacterium. Urease and its products also have additional functions. Ammonia disarranges the tissue structure; CO_2_ protects *Hp* from phagocytes, induces angiogenesis and new blood vessels formation, and incentives gastric cancer [13].

Cag-Pathogenicity Island (cagPAI) comprehends 32 genes encoding the bacterial type IV secretion system (T4SS) and the CagA protein. The role of T4SS is to translocate CagA into the epithelium. *Hp* strains expressing cagPAI are associated with chronic gastritis, peptic ulcer, and gastric cancer [14]. CagL, one more component of T4SS, cooperates with the translocation of CagA and the secretion of IL-8 from *Hp*. In vitro studies have shown that CagA induces gastric tumorigenesis in AGS cells; this result has been confirmed in chronic gastritis tissues infected with *Hp* [15].

Vacuolating Cytotoxin (VacA) is a highly pathogenic cytotoxin of *Hp*, displaying different levels of vacuolating activity associated with different genotypes. The genotype s1/m1 has high vacuolating activity; the s1/m2 has intermediate vacuolating activity, and the s2/m2 has no vacuolating activity. Patients infected with *Hp* strains expressing VacA with s1 or m1 have an increased risk of developing gastric cancer. An independent study proposes VacA as a biomarker for the prediction of peptic ulcers and gastric cancer [16]. Strong antibody response to VacA is associated with the risk of colorectal cancer [17].

In conclusion, the unique properties of this bacterium remain high mutation rate (10^−6^ per site per year) [18] and recurrent recombination during mixed infections. These properties confer to *Hp* a level of genetic diversity so high that almost every infected individual carries a distinctive strain [18].

## 4. Epigenetic and Genetic Interactions

**Figure 2:**
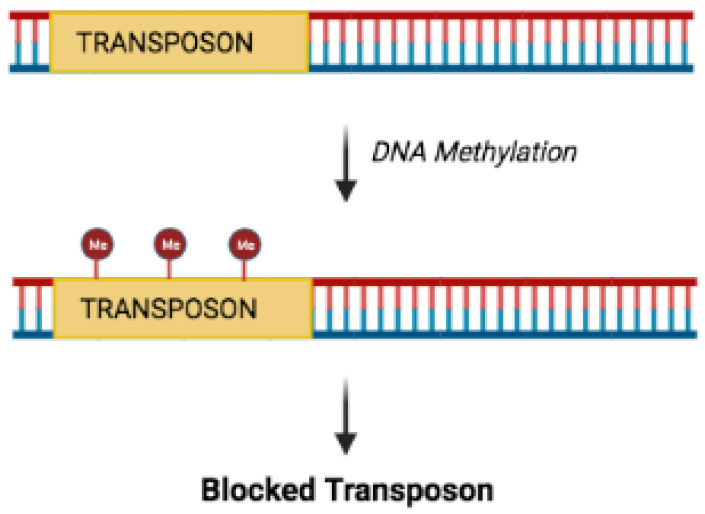
Methylation inhibits transposon mobility. Red circles: methylated transposon.

We start this section by reminding the reader that the DNA in the cells is protected by several proteins, DNA and RNA molecules, which collectively form the chromatin. In addition to gene expression, the state of chromatin also influences mutation or recombination rates of genes [19] and the movement of transposons (DNA sequences that can be inserted as a whole in the genome, creating or reversing mutations) (Figure 2) [20].

**Figure 3:**
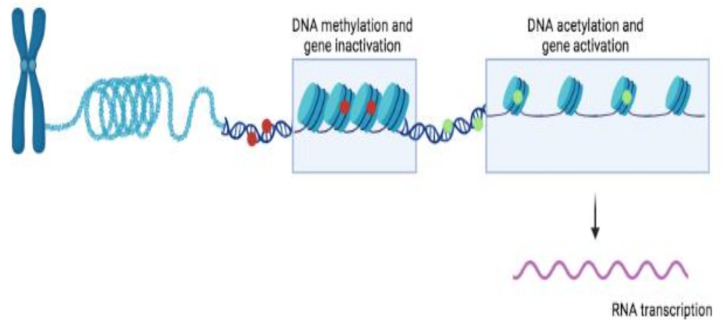
Epigenetic information at a molecular level. DNA methylation (red circles) results in close-packed nucleosomes, inhibiting transcription factors. DNA acetylation (green circles) results in loose-packed nucleosomes, favoring transcription factors.

Methylated DNA is usually associated with compact chromatin and inactive genes. Therefore, DNA is more likely to change in regions where genes are active (Figure 3). 

**Figure 4:**
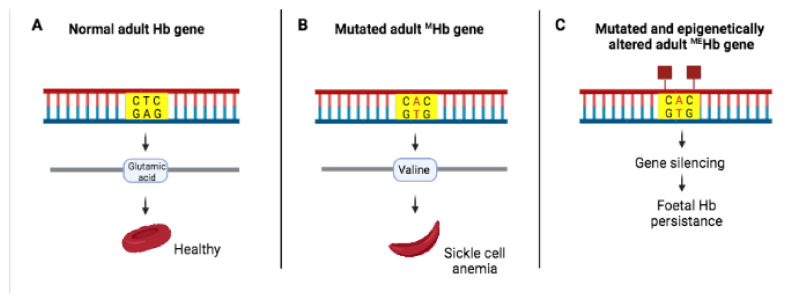
Epigenetic marks can silence mutations. (**A**) Normal adult hemoglobin (Hb) gene, encoding normal Hb protein. (**B**) Mutated adult hemoglobin (^M^Hb) gene, encoding the mutated Hb protein with the glutamic acid at position β6 replaced by valine. (**C**) Epigenetic marks (red squares) silence mutated adult ^M^Hb gene, inhibiting gene expression while fetal Hb persists.

Methylation can also silence mutations (Figure 4).

The next question to discuss is whether epigenetic changes influence diseases. As will be shown later, there is convincing evidence that the development of some forms of cancer are attributable to both genetics and epigenetics [21]. The first sign of cellular abnormality is the presence of epimutations, which methylate the regulatory regions of genes whose products normally keep DNA repaired and the cell healthy. As a result of epimutations, the damaged DNA accumulates, and cells start dividing out of control. Cancer epimutations are potentially reversible. This property is being used with success to find drugs that revert epigenetic changes; detect cancer at an earlier stage; arrest tumor growth, or monitor its progression [22,23]. In addition to cancer, epigenetics also influences infectious diseases (see below) and metabolic diseases (type 2 diabetes, obesity, cardiovascular diseases) [24,25].

## 5. Epigenetic Regulation of Gene Expression in *Hp*

In addition to high rates of mutation and recombination, *Hp* has evolved phase variation, a mechanism that provides rapid and reversible on/off switching of gene expression. Phase variation generally refers to tandem mutations in the promoter of genes encoding surface-expressed virulence genes [26]. The random switching of these genes generates phenotypically diverse subpopulations that favor the rapid adaptation of bacteria to a new niche and their evasion from the host immune response [27]. While phase variation is associated with genes encoding surface structures, several bacterial species, including *Hp,* have DNA methyltransferase genes (*modH* genes) associated with type III restriction-modification (R-M) systems, which protect the bacterial host against invasion by foreign DNA [28], without influencing recombination and consequently allelic substitution [29]. The finding that *Hp* contains many types II and type III R-M systems suggested that these methyltransferases might have functions other than preventing invasion from foreign DNA. One of these functions turned out to be the regulation of gene expression by an epigenetic mechanism, which uses the *modH* genes. The *Hp modH* gene has 17 distinct alleles, with *ModH3* and *ModH5* often detected in clinical bacterial isolates [30]. Of the genes regulated by *ModH5*, two encode the flagella (*FlaA* and *Flik*), essential to colonize the human stomach, and one (*Flik*) to evade TLR5 [31]. The gene encoding the outer membrane protein HopG is also under the control of *ModH5* [32]. Bacterial adherence mediated by the HopG protein is required for the colonization of the gastric epithelium by *Hp* [33]. The same protein has been considered as a potential vaccine to prevent *Hp* infection [34,35].

Methylation studies of several *Hp* strains have shown that every strain carries a different set of R-M systems and consequently highly different methylomes [36]. Despite the diversity of methylation patterns, a small number of targets are methylated in most (97%) of the strains [37]. The highly conserved *Hp* gene *m5C MTase* (*JHP1050*) regulates the expression of 225 genes in the strain J99 and 29 in the strain BCM-300 [38]. Transcriptome comparisons of two *Hp* wild-type strains and their *JHP1050*-knocked mutants demonstrate that *JHP1050* has a strong impact on the *Hp* transcriptome. In particular, methylation of GCGC sequences affects metabolic pathways, competence, and adherence to gastric epithelial cells. Further, methylation of GCGC overlapping the promoter plays a role in gene expression, while the regulatory effects of methylated sites outside of the promoter might be indirect [38].

Natural transformation is the process by which bacteria can spontaneously take up, integrate, and again expel exogenous DNA. This process is a source of genetic diversity and evolutionary flexibility for bacteria. *Hp* uses this tool very cleanly: to control the host immune response and, at the same time, its own pathogenicity and thus optimize host colonization. The *Hp* strain 26,695 contains only one N4 methylcytosine base (m4C). To understand the function of m4C, this methyltransferase was deleted. The m4C-deleted strain 26,695 displayed a reduced ability to adhere to the host AGS cells and induce inflammation and apoptosis. In addition, loss of m4C altered the expression of 102 genes, mainly involved in virulence and ribosome assembly, such as *CagA*, *CagZ*, catalase (*KatA*), superoxide dismutase (*SOD*), *GroES*, and the housekeeping gene *hp1541* involved in antibiotic sensitivity and recombination [39]. In conclusion, this study is the first to demonstrate that m4C acts as a global regulator of the epigenetic gene expression in *Hp*. This study explains how the spontaneous deletion and re-acquisition of m4C leads to the generation of a mixed population, essential to evade the host immune response and, at the same time, to fine regulate its pathogenicity. All this, deleting and reacquiring m4C.

## 6. *Hp*, *Epstein–Barr* Virus Co-Infection and Epigenetics

Viral infections contribute to 15–20% of all human cancers [39,40,41]. *Hp* and Epstein–Barr virus (EBV) are both oncogenic and induce chronic inflammation, which promotes gastric cancer [41]. In addition, *Hp* and EBV can establish a lifelong infection of the host [42]. Quantitative PCR demonstrated that exposure of EBV-infected gastric cells (NC1-N87) for 48 h to *Hp* increases the proliferation of the EBV-infected cells and the expression level of DNA methyltransferases (MTs), which silence the tumor suppressor genes (TSGs) controlling many pathways associated with gastric cancer (cell cycle, apoptosis, and DNA repair) [43]. When the experiment described above was repeated using cell-free *Hp* culture supernatant, the proliferation of the EBV-infected cells, increased expression levels of MTs and silencing of TSGs were again detected. This result indicates that the oncogenic activity of *Hp* is due to a soluble bacterial component. When the experiment was repeated using a CagA negative *Hp* strain, the yield of virions was similar to that obtained using EBV alone. This last experiment demonstrates that the cooperation between *Hp* and EBV is imputable to the bacterial cytotoxin CagA.

## 7. Epigenetic Markers and Cancer

Since genetics and epigenetics both contribute to gastric cancer, the detection of epigenetic markers associated with gastric cancer is precluded. However, while genetic mutations alter the DNA sequences, methylation leaves DNA sequences unaltered. This divergence offers the opportunity to identify epigenetic markers associated with gastric cancer, which is most useful for an early diagnosis and the development of new therapies against gastric cancer. Forkhead box (Fox) genes are transcriptional regulators controlling metabolism, cell differentiation, cell proliferation, and apoptosis. The promoter of the Fox gene FOXO3 is the only region displaying increased methylation in mice infected with *Hp* and in human gastric samples from patients infected with *Hp* [44]. Demethylation of a panel of gastric cancer cell lines with the DNA methyltransferase 5-aza-2′-deoxycytidine reactivates FOXD3 expression. Once reactivated, FOXD3 inhibits gastric cancer cell proliferation and reduces the growth of tumors in mice infected with *Hp* by promoting tumor cell apoptosis [42]. The same study shows that activation of FOXD3 induces the transcription of the genes *CYF1P2* and *RARB*, both regulating cell death under the control of *FOXD3*. Further, using a mouse model of *Hp* infection, human gastric cancer biopsies, and data from cell line studies, the authors of the above seminal study demonstrated that *FOXD3* is a tumor suppressor gene methylated by *Hp* and established FOXD3 promoter methylation by *Hp* as an early epigenetic marker.

Some *Hp* virulence factors contribute to gastric cancer development. The cag pathogenicity island (cagPAI) encodes a type IV secretion system (T4SS), which transfers CagA, peptidoglycan, and bacterial DNA into host cells [45]. CagA is an oncoprotein, which activates and interacts with multiple factors associated with carcinogenesis [46]. CagA activity is reinforced by the inflammation induced by *Hp* and from the interaction with the *Hp*-vacuolating cytotoxin A (VacA) [47]. Further, in EBV-coinfected gastric epithelial cells, CagA-positive *Hp* strains benefit from the oncogenic activity of CagA and the gastric microbiota [48]. In conclusion, the multi-layered activity of CagA makes *Hp* essential to start carcinogenesis but is no longer necessary once gastric cancer is triggered. As expected, *Hp* strains expressing VacA and CagA are highly virulent and cause particularly severe forms of gastric diseases. At present, it is not known whether these factors are involved with epigenetics. The hit-and-run model of carcinogenesis asserts that an infectious agent induces carcinogenesis soon after infection and directly after its presence is no longer necessary [49]. *Hp* infection and CagA activity represent a paradigm for the hit-and-run model.

## 8. *Hp* Eradication, Epigenetics, and Cancer

The virulence factors of *Hp* (VacA and CagA) alter several pathways of gastric epithelial cells [50]. Chronic inflammation and mitochondrial damage [51,52], along with reactive oxygen species (ROS) and nitric oxide (NO) associated with *Hp* infection, induce aberrant epigenetic alteration of the gastric mucosa, the main cause of gastric cancer [53]. Actually, the genes *CDKN2A*, *CDH1*, and *RUNX3* found in gastric cancer and in precancerous lesions of *Hp*-infected patients [54] inactivate the tumor-suppressor genes. Further, animal experiments have shown that inhibition of aberrant DNA methylation often prevents gastric cancer development [55]. Genetic factors also contribute to gastric cancer. Mutations associated with gastric cancer have been identified in the genes *TP53*, *CDH1*, *ARIDIA*, and *RHOA* [3,56,57]. However, mutations account for 15% of all gastric cancer cases [3]. Actually, the inactivation of the cancer-suppressor genes *hMLH1* and *CDH1* frequently results from aberrant DNA methylation rather than mutation.

Eradication of *Hp* following resection of early cancer prevents the development of gastric cancer [58]. However, the eradication of *Hp* does not prevent the development of de novo cancer [59]. *Hp* eradication prevents cancer development only in patients without premalignant lesions (atrophy, intestinal metaplasia (IM), and dysplasia) [60]. Once IM is present, there is a six-fold increased risk of gastric cancer [61]. These results indicate that the molecular damage is “imprinted” in the genome of premalignant lesions. DNA methylation in premalignant lesions marks the point of “no return”.

The residual epigenetic imprints favor permanent changes in gene expression. Given that cancer originates from a cell that has the potential to divide, bacterial reprogramming very likely occurs in stem cells. The use of mitochondrial DNA (mtDNA) mutations as a marker of clonal expansion confirmed that the mutation originally occurs in a single stem cell that spreads by binary fission until a loan of mutated epithelial cells develops, leading to tumor development [62]. This experiment also proves the role of mtDNA mutation in the development of gastric cancer [63]. The mtDNA mutations (AT > GC and GC > AT transitions) frequently occur in *Hp*-positive patients with gastric cancer [64], as confirmed in vivo on gastric biopsies from *Hp-*infected patients with chronic gastritis [65] and on the gastric mucosa of *Hp-*infected mice at 12 and 18 months from infection [66]. By inducing mutation in the mitochondrial genome, *Hp* impairs oxidative phosphorylation metabolism, increasing ROS production and mtDNA damage. In conclusion, the oncogenic properties of *Hp* result from several components: the genotoxic effect of its virulence factors and genetic and epigenetic factors. Indirectly *Hp* also modulates gene expression by downregulating transcription factors and DNA repair gene expression [67].

## 9. Epigenetics: A Resource for Bacterial Survival

In this section, we briefly diverge from *Hp* to remind the reader that many bacteria, other than *Hp,* manipulate the host epigenetically [68]. We mention a few: *E. coli* suspected of predisposing to bladder carcinoma risk [69] and intestinal bacteria to colon cancer [70]. *Mycobacterium tuberculosis*, methylates several genes of the host immune response with its protein RV1988 [71]. *Listeria monocytogenes* reaches the same objective using a different approach: it avoids its clearance modulating the activation of the host T-cells. This topic is discussed extensively in [68].

Several pathways, including Wnt, Hedgehog, and Notch, are common to embryogenesis and cancer. These pathways are conserved during mammalian evolution. To form the fetus, the embryogenesis cells must change configuration, in order to reach their location. Thus, embryogenesis cells undergo epithelial–mesenchymal transition (EMT). The EMT phenotype includes enhanced migratory capacity, invasiveness, resistance to apoptosis, and loss of the E-chatherin, a protein implicated in the development of gastric carcinoma [72]. A subset of patients with medulloblastoma carry mutations in the *SUFU* gene. Loss of function of this gene has been claimed to upregulate the Hedgehog and Wnt pathways, which in turn dysregulate cellular proliferation [73]. The case described is one of many attributable to genes involved in both embryogenesis and cancer [72]. It is difficult to anticipate the therapeutic utility of studying this gene subset. However, thanks to what we know about the epigenetic memory of cells, we can appreciate how much the beginning and the end of life have in common.

## 10. Conclusions

Many of the host genes targeted by *Hp* remain methylated after its eradication, and the oncogenic properties of *Hp* derive from different sources: the toxic effects of bacterial virulence factors; mutations in the mitochondrial genome; altered oxidative phosphorylation metabolism; and increased ROS production. However, the most important oncogenic factor of *Hp* remains the aberrant epigenetic alteration of the gastric mucosa. We remind the reader that, in addition to *Hp*, several more bacteria manipulate the host epigenetically.
